# Ribovore: ribosomal RNA sequence analysis for GenBank submissions and database curation

**DOI:** 10.1186/s12859-021-04316-z

**Published:** 2021-08-12

**Authors:** Alejandro A. Schäffer, Richard McVeigh, Barbara Robbertse, Conrad L. Schoch, Anjanette Johnston, Beverly A. Underwood, Ilene Karsch-Mizrachi, Eric P. Nawrocki

**Affiliations:** 1 Cancer Data Science Laboratory, National Cancer Institute, National Institutes of Health, Bethesda, MD 20892 USA; 2 National Center for Biotechnology Information, National Library of Medicine, National Institutes of Health, Bethesda, MD 20894 USA

**Keywords:** Ribosomal RNA, Annotation, Alignment, ncRNA

## Abstract

**Background:**

The DNA sequences encoding ribosomal RNA genes (rRNAs) are commonly used as markers to identify species, including in metagenomics samples that may combine many organismal communities. The 16S small subunit ribosomal RNA (SSU rRNA) gene is typically used to identify bacterial and archaeal species. The nuclear 18S SSU rRNA gene, and 28S large subunit (LSU) rRNA gene have been used as DNA barcodes and for phylogenetic studies in different eukaryote taxonomic groups. Because of their popularity, the National Center for Biotechnology Information (NCBI) receives a disproportionate number of rRNA sequence submissions and BLAST queries. These sequences vary in quality, length, origin (nuclear, mitochondria, plastid), and organism source and can represent any region of the ribosomal cistron.

**Results:**

To improve the timely verification of quality, origin and loci boundaries, we developed Ribovore, a software package for sequence analysis of rRNA sequences. The ribotyper and ribosensor programs are used to validate incoming sequences of bacterial and archaeal SSU rRNA. The ribodbmaker program is used to create high-quality datasets of rRNAs from different taxonomic groups. Key algorithmic steps include comparing candidate sequences against rRNA sequence profile hidden Markov models (HMMs) and covariance models of rRNA sequence and secondary-structure conservation, as well as other tests. Nine freely available blastn rRNA databases created and maintained with Ribovore are used for checking incoming GenBank submissions and used by the blastn browser interface at NCBI. Since 2018, Ribovore has been used to analyze more than 50 million prokaryotic SSU rRNA sequences submitted to GenBank, and to select at least 10,435 fungal rRNA RefSeq records from type material of 8350 taxa.

**Conclusion:**

Ribovore combines single-sequence and profile-based methods to improve GenBank processing and analysis of rRNA sequences. It is a standalone, portable, and extensible software package for the alignment, classification and validation of rRNA sequences. Researchers planning on submitting SSU rRNA sequences to GenBank are encouraged to download and use Ribovore to analyze their sequences prior to submission to determine which sequences are likely to be automatically accepted into GenBank.

## Background

In 1977, Carl Woese and George Fox proposed the *Archaebacteria* (later renamed *Archaea*) as a third domain of life distinct from *Bacteria* and *Eukaryota* based on analysis of small subunit ribosomal RNA (SSU rRNA) oligonucleotide fragments from 13 microbes [1]. The use of SSU rRNA to elucidate phylogenetic relationships continued and dramatically expanded in the late 1980s when Norm Pace and colleagues developed a technique to PCR amplify rRNA genes from potentially unculturable microbes in environmental samples by targeting so-called *universal primer sites* [2]. The technique was later refined by Pace and others including Ward, Weller [3] and Giovanonni et al. [4]. Environmental studies targeting SSU rRNA as a *phylogenetic marker gene* that seek to characterize the diversity of life in a given environment have remained common ever since, and consequently there are now millions of prokaryotic SSU rRNA sequences in public databases. When rRNA sequences are submitted to public databases, such as GenBank, it is important to do quality control, so that subsequent data analyses are not misled by errors in sequencing and sequence annotation. Because rRNA gene sequences do not code for proteins, but have been studied so extensively, specialized checks for correctness and completeness are feasible and desireable. The focus of this paper is the description of Ribovore, a software package for validating incoming rRNA sequence submissions to GenBank and for curating rRNA sequence collections.

SSU rRNA was initially chosen by Woese and Fox for inferring a universal phylogenetic tree of life because it existed in all cellular life, was large enough to provide enough data (about 1500 nucleotides (nt) in *Bacteria*), and had evolved slowly enough to be comparable across disparate groups [5]. The first environmental surveys targeted SSU rRNA, but studies targeting LSU rRNA, which is roughly twice as long as SSU rRNA, followed soon after [6, 7]. These types of analyses eventually began to target eukaryotes, especially *Fungi* [8].

In eukaryotes, the 5.8S rRNA gene is surrounded by two internal transcribed spacers (ITS1 and ITS2). This region is sometimes collectively referred to as the ITS region and it has been selected as the primary fungal *barcode* since it has the highest probability of successful identification for the broadest range of *Fungi* [9]. However, the LSU rRNA gene [10] is a popular phylogenetic marker in certain fungal groups [9]. In general, the nuclear SSU rRNA has poor species-level resolution in most *Fungi* and other eukaryote taxonomic groups [9, 11], but remains useful at species-level phylogenetic inference in some rapid evolving groups such as the diatoms [12]. Species identification in protists takes a two-step barcoding approach, which use the $\sim 500$ bp variable V4 region of the SSU rRNA gene as a variable marker and then use a group-specific barcode for species-level assignments, some of which include the LSU rRNA gene and ITS region [11].

Specialized analysis tools and databases have been developed to help researchers analyze their rRNA sequences. Many of these specialized tools are based on comparing sequences to either profile hidden Markov models (profile HMMs) or covariance models (CMs). CMs are profile stochastic context-free grammars, akin to profile HMMs of sequence conservation [13, 14], with additional complexity to model the conserved secondary structure of an RNA family [15–17]. Like profile HMMs, CMs are probabilistic models with position-specific scores, determined based on the frequencies of nucleotides at each position of the input *training* alignment used to build the model. Unlike HMMs, CMs also model well-nested secondary structure, provided as a single, fixed consensus secondary structure for each model and annotated in the input training alignment. A CM includes scores for each of the possible 16 (4x4) basepairs for basepaired positions and both paired positions are considered together by scoring algorithms.

The incorporation of secondary structure has been shown to significantly improve remote homology detection of structural RNAs [18], and for SSU rRNA considering structure has been shown to offer a small improvement to alignment accuracy versus profile HMMs [19, 20]. For eukaryotes, where SSU and LSU rRNA sequences are often more divergent at the sequence level than for *Bacteria* and *Archaea*, harnessing structural information during alignment may be more impactful.

The specialized tools for rRNA sequence analysis include: databases, some of which are integrated with software, rRNA prediction software, and multiple alignment software. The integrated and highly curated databases include the ARB workbench software package for rRNA database curation [21], the Comparative RNA Website (CRW) [22], the Ribosomal Database Project (RDP) [23, 24], and the Greengenes [25], and Silva [26] databases. These databases differ in their scope and methodology. CRW contains tens of thousands of sequences and corresponding alignments of SSU, LSU and 5S rRNA from all three domains as well as from organelles, along with secondary structure predictions for selected sequences. Greengenes, which is seemingly no longer maintained as its last update was in 2013, includes SSU rRNA sequences for *Bacteria* and *Archaea*, but not for *Eukarya*, nor does it contain any LSU rRNA sequences. RDP also includes SSU rRNA for *Bacteria* and *Archaea*, as well as fungal LSU rRNA and ITS sequences, but no other LSU sequences. Silva, which split off from the ARB project starting in 2005 [27], includes bacterial, archaeal and eukaryotic (fungal and non-fungal) SSU and LSU rRNA sequences. RDP includes more than 3 million SSU rRNA and 125,000 fungal LSU rRNA sequences as of its latest release (11.5), and Silva includes more than 9 million SSU rRNA and 1 million LSU rRNA sequences (release 138.1).

Available rRNA prediction software packages include RNAmmer [28], rRNAselector [29], and barrnap(https://github.com/tseemann/barrnap) all of which use some version of the profile HMM software HMMER [30] to predict the locations of rRNAs in contigs or whole genomes.

Both RDP and Silva make available multiple alignments of all sequences for each gene and taxonomic domain, and all include several sequence analysis tools for tasks such as classification. The alignment methodology differs: Silva uses SINA, which implements a graph-based alignment algorithm that computes a sequence-only based alignment of an input sequence to one or more similar sequences selected from a fixed reference alignment [31]. RDP uses Infernal [32], which computes alignments using CMs.

Per-domain CMs for SSU and LSU rRNA are freely available in the Rfam database, a collection of more than 3900 RNA families each represented by a consensus secondary structure annotated reference alignment called a *seed* alignment and corresponding CM built from that alignment [33]. Rfam includes five full length SSU and four full length LSU rRNA families and CMs. Although RDP uses CMs for rRNA alignment, the CMs are not from Rfam. Users can download and use Rfam CMs to annotate their own sequences using Infernal, thus offering a distinct strategy from Silva or RDP for rRNA analysis.

The Rfam database includes a model (RF02542) for SSU rRNA from *Microsporidia*, a phylum of particular interest within the kingdom of *Fungi*. More than 30 years ago, Woese and colleagues discovered that *Microsporidia* have a distinctive ribosome that is smaller and more primitive than the ribosomes of most if not all other eukaryotes [34]. Recently, Barandun and colleagues presented the first crystal structure of the ribosome of *Microsporidia*, confirming that both the SSU and LSU rRNA are smaller than in other *Fungi* [35]. Most of the sequence analysis and curation to date in *Microsporidia* has focused on SSU rather than LSU rRNA.

### GenBank processing of rRNA sequences

Data submitted to GenBank are subject to review by NCBI staff to prevent incorrect data from entering NCBI databases. Over the past three decades, personnel called *GenBank indexers* have spent a large proportion of their time validating incoming submissions of thousands to millions of rRNA sequences due to the large number of rRNA sequences generated in phylogenetic and environmental studies. Similarity searches with blastn have been used to compare submitted rRNA sequences against databases of trusted, high-quality rRNA sequences for prokaryotic 16S and 23S, eukaryotic 18S and 28S, and chloroplast-specific sequences. However, these databases were not comprehensive before curation with Ribovore began. The blastn query results were a primary source of evidence used to determine if rRNA sequences would be accepted to GenBank or not. Prior to the Ribovore project, suitable blastn databases did not exist for validating submissions of eukaryotic SSU rRNA or LSU rRNA sequences, making checking for those genes especially difficult and time-consuming.

Starting in 2016, a system with predefined criteria for per-sequence blastn results was deployed at NCBI; submissions in which all sequences met those criteria have been automatically accepted into GenBank without any indexer review. Ribovore-based tests began being used in conjunction with or instead of blastn-based tests for some submissions in this system in June 2018. Although the engine inside the pre-2018 validation system, BLAST, is freely available and portable, the system as a whole was internal to GenBank and not portable, preventing researchers who wish to submit sequence data to GenBank (henceforth, called “submitters”) from replicating the tests on their local computers.

For rRNA sequences as well as other sequences of high biological interest, GenBank indexers and other NCBI personnel want to carry out two related and recurrent processes: quick identification of which submitted sequences should be accepted into GenBank, and the construction of non-redundant collections of trusted, full length sequences that have no or few errors. The second problem is the motivation behind the entire RefSeq project [36]. Towards addressing the first problem, the development of an alternative sequence validation system for rRNA included four design goals offering potential improvements over the existing system. First, the system should be as deterministic and as reproducible as possible in deciding whether sequences are accepted or not, which we refer to as *passing* (accepted) or *failing* (not accepted), allowing submissions with zero failing sequences to be automatically added to GenBank without the need for any manual GenBank indexer intervention. It is unavoidable that automated decisions about acceptance can change over time because various inputs to the system, such as the NCBI taxonomy tree, change over time. Second, the system should be available as a standalone tool that submitters can run on their sequences prior to submission, saving time for both the GenBank indexers and submitters. Third, the system should be general enough to facilitate extension to additional taxonomic groups and rRNA genes. Fourth, the system should be capable of increasing the stringency of tests for quality and adding tests to avoid redundancy to enable producing collections of high quality non-redundant sequences for other applications, such as serving as blastn databases.

Because none of the existing databases or specialized rRNA tools listed above address all of these design goals, we implemented the freely available and portable Ribovore software package for the analysis of SSU rRNA and LSU rRNA sequences from *Bacteria*, *Archaea*, and *Eukarya* as well as mitochondria from some eukaryotic groups. Ribovore includes several programs designed for related but distinct tasks, each of which has specific rules dictating whether a sequence passes or fails based on deterministic criteria described in detail in the Implementation section and in the Ribovore documentation. rRNA_sensor is a simplified, standalone version of the previous blastn-based system that is more portable and faster for bacterial and archaeal SSU rRNA than the previous system owing to a smaller blastn target database constructed by removing redundancy from the pre-existing blastn database. ribotyper is similar to rRNA_sensor but compares each input sequence against a library of profile HMMs and/or CMs offering an alternative, and in some cases, more powerful approach than the single sequence-based blastn algorithm. Additionally, ribotyper can be used to validate the taxonomic domain each sequence belongs to because it compares a set of models from different taxonomic groups against each sequence. To take advantage of both single-sequence and profile-based approaches, and partly to ease the transition from the previous blastn-based system towards profile-based analysis, we implemented ribosensor that runs both rRNA_sensor and ribotyper and then combines the results. Up to this point, rRNA_sensor and ribotyper are deliberately designed to accept both partial and complete sequences of moderate quality or better. To more selectively identify full-length rRNA sequences that extend up to, but not beyond the gene boundaries, we implemented riboaligner which runs ribotyper as a first pass validation, and then creates multiple alignments and selects sequences that pass based on those alignments. Finally, to make Ribovore capable of generating datasets of trusted sequences from different taxonomic groups for wider use by the community, we developed ribodbmaker, which chooses a non-redundant set of high-quality, full-length sequences based on a series of tests. The pipeline of tests includes some specific to rRNA, including analysis by ribotyper and riboaligner, some more general tests, such as counting ambiguous nucleotides and vector contamination screening, and some tests that require connection to the NCBI taxonomy database to validate the taxonomy assignment of sequences.

## Implementation

Ribovore is written in Perl and available at https://github.com/ncbi/ribovore. The Ribovore installation procedure also installs the program rRNA_sensor, which is described here as well. The rRNA_sensor program includes a shell script and Perl scripts and is available at https://github.com/aaschaffer/rRNA_sensor. These packages use existing software as listed in Table 1.

**Table 1:** Software packages and libraries used within Ribovore v1.0

Software and website	Used within	Purpose in Ribovore
Sequip v0.08 github.com/nawrockie/sequip	All Ribovore programs	Option handling, output file handling and other utilities
Infernal v1.1.4^*^ github.com/EddyRivasLab/infernal	All Ribovore programs	Build and use profile HMMs and CMs to classify, validate and align rRNA sequences
BLAST+ v2.11.0 ftp.ncbi.nlm.nih.gov/blast/ executables/blast+/2.11.0	Ribodbmaker	Build BLAST databases and validate rRNA sequences
VecScreen_plus_taxonomy v0.17 github.com/aaschaffer/ vecscreen_plus_taxonomy	Ribodbmaker	Screen for vector contamination
GNU time (not required)	All Ribovore programs if -p option is used	Determine running time

^*^: the esl-cluster executable from Infernal v1.1.2, which is absent in v1.1.4, is also installed and used within Ribovore

**Fig. 1:**
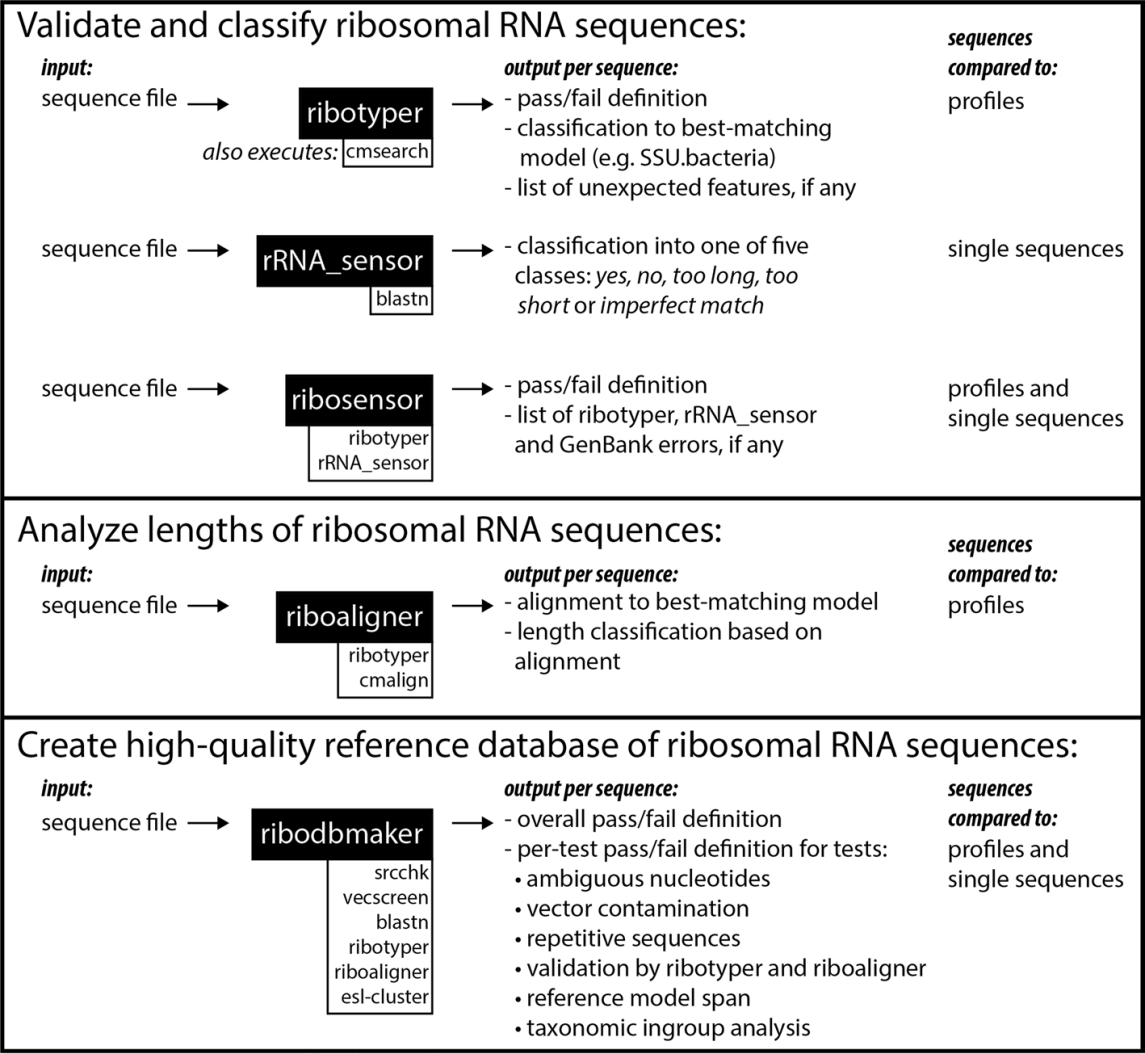
Schematic summarizing the use cases for the four Ribovore programs and rRNA_sensor. Programs listed in white boxes underneath the black boxes are important external programs executed from within the program in the attached black box

Each of the four Ribovore programs takes as input two command-line arguments: the path to an input sequence file in FASTA format and the name of an output directory to create and store output files in. Command-line options exist to change default parameters and behavior of the programs in various ways. The options as well as example usage can be found as part of the source distribution and on GitHub in the form of markdown files in the Ribovore documentation subdirectory (e.g. https://github.com/ncbi/ribovore/blob/master/documentation/ribotyper.md). Central to each of the scripts is the concept of sequences passing or failing. If a sequence meets specific criteria, many of which are changeable with command-line options, then it will pass and otherwise it will fail, as discussed more below. An overview of the four Ribovore programs and rRNA_sensor is shown in Fig. 1.

### rRNA_sensor

The rRNA_sensor program compares input sequences to a blastn database of verified rRNA sequences using blastn. The program takes nine command-line arguments specified in Table 2. Each input sequence is classified into one of five classes based on its length and blastn results. A sequence is classified as *too long* or *too short* if its length is greater than the maximum length or less than the minimum length specified in the command by the user. To allow partial sequences and flexibility in the length, GenBank indexers were typically using a length interval of [400,2500] nt for prokaryotic 16S SSU rRNA. Empirical analysis shows that more than 99.8% of the full-length validated prokaryotic sequences have lengths in the range [900,1800], so this narrower range is recommended if one wants to check that sequences are typically full-length sequences.

**Table 2:** Command-line arguments for rRNA_sensor

Argument index	Argument name	Description
1	Min_length	Lower bound on sequence length
2	Max_length	Upper bound on sequence length
3	Seq_file	Input sequence file in FASTA format
4	Output_file_name	Name for summary output file
5	Min_id_percentage	Lower bound on percent identity
6	Max_Evalue	Upper bound on E-value
7	Nprocessors	Number of threads for blastn
8	Output_dir	Output directory path
9	Blastdb	Blastn database

Sequences within the allowed length range are classified as either *no* if there are zero blastn hits, *yes* if they have at least one blastn hit that has an E-value of 1e−40 or less and a percent identity of 80% or more, or *imperfect*_*match* if there is at least one hit but the E-value or percent identity thresholds are not met for any hits. Sequences that are too long are probably either incorrect or containing extra flanking sequence that should be trimmed, while sequences that are too short may be valid partial sequences. The other tests based on quality of blastn matches codify the tests that GenBank indexers were doing internally before rRNA_sensor was implemented. Submitted sequences of a suitable length now classified as *no* would have been rejected in the past framework; sequences now classified as *yes* would have been accepted into GenBank in the past framework. In the current testing framework, rRNA_sensor is used as part of the ribosensor program as described below, not by itself.

There are two target blastn databases included with rRNA_sensor, one for prokaryotic 16S SSU rRNA and one for eukaryotic 18S SSU rRNA. The prokaryotic database includes 1267 sequences, 1205 of which are bacterial and the remaining 62 are archaeal. The eukaryotic database includes 1091 sequences. Additional, user-created blastn databases can also be used with the program. The prokaryotic database was updated most recently on June 29, 2017 by filtering and clustering the pre-existing database of 18,816 sequences used by GenBank indexers for 16S SSU rRNA analysis. One could repeat the same procedure with the larger version of the 16S SSU rRNA database described in Results. The initial database was filtered to remove 26 sequences outside the length range [900,1800]. The remaining 18,790 sequences were clustered using UCLUST [37] so that the surviving sequences were no more than 90% identical, leaving 1267 sequences. The eukaryotic 18S SSU rRNA database of 1091 sequences was updated most recently on September 27, 2018 by running version 0.28 of the Ribovore program ribodbmaker on an input set of 579,279 GenBank sequences returned from the eukaryotic SSU rRNA E-utilities (eutils) query provided in Results and discussion with command-line options --skipfribo1 --model SSU.Eukarya --ribo2hmm.

### Ribotyper

The ribotyper program is also designed to validate ribosomal RNA sequences but it differs from rRNA_sensor in the method of sequence comparison and the taxonomic breadth over which it applies. Instead of using blastn, ribotyper uses a profile HMM and optionally a covariance model (CM) to compare against input sequences. The profile HMM and CMs were built either from Rfam rRNA seed alignments (see Table 3) or from alignments created specifically for Ribovore by the authors for taxonomic groups not covered by the Rfam models.

**Table 3:** Profile models used by Ribovore

Model name	Gene	Taxonomy group	#Seqs	Length	Rfam
SSU_rRNA_archaea	SSU rRNA	Archaea	86	1477	RF01959
SSU_rRNA_bacteria	SSU rRNA	Bacteria	99	1533	RF00177
SSU_rRNA_eukarya	SSU rRNA	Eukarya	91	1851	RF01960
SSU_rRNA_microsporidia	SSU rRNA	Euk-Microsporidia	46	1312	RF02542
LSU_rRNA_archaea	LSU rRNA	Archaea	91	2990	RF02540
LSU_rRNA_bacteria	LSU rRNA	Bacteria	102	2925	RF02541
LSU_rRNA_eukarya	LSU rRNA	Eukarya	88	3401	RF02543
SSU_rRNA_mitochondria_metazoa	SSU rRNA	Mito-Metazoa	83	954	–
SSU_rRNA_mitochondria_amoeba	SSU rRNA	Mito-Amoeba	2	1861	–
SSU_rRNA_mitochondria_chlorophyta	SSU rRNA	Mito-Chlorophyta	2	1200	–
SSU_rRNA_mitochondria_fungi	SSU rRNA	Mito-Fungi	4	1603	–
SSU_rRNA_mitochondria_kinetoplast	SSU rRNA	Mito-Kinetoplast	3	624	–
SSU_rRNA_mitochondria_plant	SSU rRNA	Mito-Plant	4	1951	–
SSU_rRNA_mitochondria_protist	SSU rRNA	Mito-Protist	2	1677	–
SSU_rRNA_chloroplast	SSU rRNA	Chloroplast	94	1488	–
SSU_rRNA_chloroplast_pilostyles	SSU rRNA	Chloroplast	1	1531	–
SSU_rRNA_cyanobacteria	SSU rRNA	Bac-Cyanobacteria	49	1487	–
SSU_rRNA_apicoplast	SSU rRNA	Euk-Apicoplast	3	1463	–

’#seqs’ is the number of sequences in the multiple alignment used to build the model. ’length’ is the number of reference model positions. Abbreviations in ’taxonomy group’ column: ’Bac’ is Bacteria, ’Euk’ is Eukarya and ’Mito’ is Mitochondria

Sequence processing by ribotyper proceeds over two main stages. In stage 1, each sequence is compared against all profiles using a truncated version of the HMMER3 pipeline [38] optimized for speed. Only the first three stages of the HMMER3 pipeline are employed to compute a score for each sequence/profile comparison but without calculating accurate alignment endpoints. For each sequence, the best-scoring model is selected and used in the second stage where the HMMER3 pipeline is used again but this time in its entirety to compute likely endpoints of high-scoring hits to each model. These two stages are very similar to the classification and coverage determination stages of the VADR software package for viral sequence annotation [39]. The results of the stage 2 comparison are then post-processed to determine if any *unexpected features* exist for each sequence. There are 16 types of unexpected features, listed in Table 4.

**Table 4:** Attributes of the 16 types of ribotyper unexpected features

Unexpected feature name	Description
NoHits^*^	No stage 1 hits above threshold to any models
UnacceptableModel^*^	Best stage 1 hit is to a model that is *unacceptable* as defined in --inaccept input file
MultipleFamilies^*^	Stage 1 hits exist to more than one family (e.g. SSU and LSU)
BothStrands^*^	Stage 1 hits above threshold exist on both strands
DuplicateRegion^*^	At least two stage 1 or 2 hits on same strand overlap
InconsistentHits^*^	Not all hits are in the same order in sequence and model coordinates
QuestionableModel^*^	Best stage 1 hit is to a model that is *questionable* as defined in --inaccept input file
MinusStrand	Best stage 1 hit is on the minus strand
LowScore	The bits per nucleotide value (total bit score divided by total length of sequence) is below threshold of 0.50
LowCoverage	Sequence coverage of all hits is below threshold of 0.86
LowScoreDifference	Difference between top two models in different domains is below 0.10 bits per position
VeryLowScoreDifference	Difference between top two models in different domains is below 0.04 bits per position
MultipleHits	There is more than one hit to the best scoring model on the same strand
EvalueScoreDiscrepancy	If hits were sorted by E-value due to --evalue, best hit has lower bit score than second best hit
TooShort*	Sequence length is less than <n> and --shortfail <n> used
TooLong*	Sequence length is greater than <n> and --shortlong <n> used

Unexpected features labelled with * in the first column are fatal by default, in that they cause a sequence to fail. UnacceptableModel and QuestionableModel can only potentially be reported if the --inaccept option is used. EvalueScoreDiscrepancy can only be reported if the --evalues option is used. TooShort and TooLong can only be reported if the --shortfail or --longfail options are used, respectively

### Ribosensor

The ribosensor program is a wrapper script that runs both ribotyper and rRNA_sensor and combines the results to determine if each sequence should pass or fail. This script was motivated partly by an effort to ease the transition for GenBank indexers between the pre-existing blastn-based system and a system based on profiles. Additionally, in some cases, the profile models in ribotyper allow some valid rRNA sequences that would fail blastn and rRNA_sensor to pass, and conversely some valid sequences pass rRNA_sensor and fail ribotyper, making a combination of the two programs potentially more accurate.

The ribosensor program can be run in one of two modes: 16S mode is the default mode and should be used for bacterial and archaeal 16S SSU rRNA sequences, and 18S mode should be used (by specifying the option -m 18S on the command-line) for eukaryotic 18S SSU rRNA. All sequences are first processed by ribotyper using command-line options --scfail --covfail --tshortcov 0.80 --tshortlen 350 to fail sequences for which LowScore and LowCoverage unexpected features are reported, and to specify that the threshold for LowCoverage is 80% for sequences of 350 nt or less. These options were selected based on results of internal testing by GenBank indexers. Next, rRNA_sensor is run, potentially up to three separate times, on partitions of the input sequence file separated based on length and using custom thresholds for each length range. Sequences that are shorter than 100 nt or longer than 2000 nt are considered too short or too long and are not analyzed. For sequences between 100 and 350 nt, a minimum percent identity of 75% and minimum coverage of 80% is enforced. For sequences between 351 and 600 nt, the minimum thresholds used are 80% identity and 86% coverage, and for sequences between 601 and 2000 nt the minimum thresholds used are 86% percent identity and 86% coverage. These thresholds can be changed via command-line options.

The results of ribotyper and rRNA_sensor are combined and each sequence is separated into one of four outcome classes depending on whether it passed or failed each program: *RPSP* (passed ribotyper and rRNA_sensor), *RPSF* (passed ribotyper and failed rRNA_sensor), *RFSP* (failed ribotyper and passed rRNA_sensor), and *RFSF* (failed both). Additionally, the reasons for failing each program are reported. For ribotyper, these are the unexpected features described above, each prefixed with a “R_” (e.g. R_MultipleFamilies). The possible errors for rRNA_sensor are listed in Table 5 and the possible errors for ribotyper are listed in Table 6. Finally, these errors are mapped to a different set of errors created for use within the pre-existing context of GenBank’s sequence processing pipeline which has its own error naming and usage conventions. This mapping is shown in Table 7. The “fails to” column is of practical importance because it indicates which errors cause a submission to not be accepted. If a submission fails to the submitter, then the submitter is expected to make corrections. In contrast, if a submission fails to indexer, then GenBank personnel review the error and determine if the submission can be corrected without further intervention by the submitter. If not, the submitter is contacted and asked for more information or to make the necessary corrections. More positively, if a submitter runs ribosensor before actually trying to submit and the submitter sees that the errors in the first seven rows and the third column of Table 7 do not occur, then, assuming the metadata for the submission are complete and valid, the submitter can have confidence that the submission to GenBank will be accepted.

**Table 5:** Descriptions of rRNA_sensor errors within ribosensor and mapping to the GenBank errors they trigger

rRNA_sensor error	Associated GenBank error	Cause/explanation
S_NoHits^*^	SEQ_HOM_NotSSUOrLSUrRNA	No hits reported (‘no’ column 2)
S_NoSimilarity^*^	SEQ_HOM_LowSimilarity	Coverage (column 5) of best blast hit is < 10%
S_LowSimilarity^*^	SEQ_HOM_LowSimilarity	Coverage (column 5) of best blast hit is < 80% (≤ 350 nt) or 86% (> 350 nt)
S_LowScore^*^	SEQ_HOM_LowSimilarity	Either id percentage below length-dependent threshold (75%, 80%, 86%) or E-value above 1e−40 (’imperfect_match’ column 2)
S_BothStrands	SEQ_HOM_MisAsBothStrands	Hits on both strands (’mixed’ column 2)
S_MultipleHits	SEQ_HOM_MultipleHits	More than 1 hit reported (column 4 > 1)

‘*’: The first four rRNA_sensor errors do not trigger GenBank errors and are ignored by ribosensor if either (a) the sequence is ’RPSF’ (passes ribotyper and fails rRNA_sensor) and the -c option is *not* used with ribosensor or (b) the sequence is ’RFSF’ (fails both ribotyper and rRNA_sensor) and R_UnacceptableModel or R_QuestionableModel ribotyper errors are also reported

**Table 6:** Descriptions of ribotyper errors within ribosensor and mapping to the GenBank errors they trigger

Ribotyper error	Associated GenBank error	Cause/explanation
R_NoHits	SEQ_HOM_NotSSUOrLSUrRNA	No hits reported
R_MultipleFamilies	SEQ_HOM_SSUAndLSUrRNA	SSU and LSU hits
R_LowScore	SEQ_HOM_LowSimilarity	Bits/position score is < 0.5
R_BothStrands	SEQ_HOM_MisAsBothStrands	Hits on both strands
R_InconsistentHits	SEQ_HOM_MisAsHitOrder	Hits are in different order in sequence and model
R_DuplicateRegion	SEQ_HOM_MisAsDupRegion	Hits overlap by 10 or more model positions
R_UnacceptableModel	SEQ_HOM_TaxNotExpectedSSUrRNA	Best hit is to model other than expected set 16S expected set: SSU.Archaea, SSU.Bacteria, SSU.Cyanobacteria, SSU.Chloroplast 18S expected set: SSU.Eukarya
R_LowCoverage	SEQ_HOM_LowCoverage	Coverage of all hits is < 0.80 (if ≤ 350 nt) or 0.86 (if > 350 nt)
R_QuestionableModel^+^	SEQ_HOM_TaxQuestionableSSUrRNA	Best hit is to a ’questionable’ model (if mode is 16S: SSU.Chloroplast)
R_MultipleHits^+^	SEQ_HOM_MultipleHits	More than 1 hit reported

‘+’: these errors errors do not trigger a GenBank error if sequence is ’RFSP’ (fails ribotyper and passes rRNA_sensor)

**Table 7:** Mapping of GenBank errors to the rRNA_sensor and ribotyper errors that trigger them

GenBank error	Fails to	Triggering rRNA_sensor/ribotyper errors
SEQ_HOM_NotSSUOrLSUrRNA	Submitter	S_NoHits^*^, R_NoHits
SEQ_HOM_LowSimilarity	Submitter	S_NoSimilarity^*^, S_LowSimilarity^*^, S_LowScore^*^, R_LowScore
SEQ_HOM_SSUAndLSUrRNA	Submitter	R_MultipleFamilies
SEQ_HOM_MisAsBothStrands	Submitter	S_BothStrands, R_BothStrands
SEQ_HOM_MisAsHitOrder	Submitter	R_InconsistentHits
SEQ_HOM_MisAsDupRegion	Submitter	R_DuplicateRegion
SEQ_HOM_TaxNotExpectedSSUrRNA	Submitter	R_UnacceptableModel
SEQ_HOM_TaxQuestionableSSUrRNA	Indexer	R_QuestionableModel^+^
SEQ_HOM_LowCoverage	Indexer	R_LowCoverage
SEQ_HOM_MultipleHits	Indexer	S_MultipleHits, R_MultipleHits^+^

There are two classes of exceptions marked by two different superscripts in the table: ‘*’: these rRNA_sensor errors do not trigger a GenBank error if: (a) the sequence is ’RPSF’ (passes ribotyper and fails rRNA_sensor) and the -c option is *not* used with ribosensor. or (b) the sequence is ’RFSF’ (fails both ribotyper and rRNA_sensor) and R_UnacceptableModel or R_QuestionableModel are also reported. ‘+’: these ribotyper errors do not trigger a GenBank error if sequence is ’RFSP’ (fails ribotyper and passes rRNA_sensor)

### Riboaligner

The riboaligner program was designed to help GenBank indexers to evaluate whether ribosomal RNA sequences are full length and do not extend past the boundaries of the gene. One application for a set of full length rRNAs is as part of the blastn database for screening and validating incoming sequences using rRNA_sensor, ribosensor or other blastn-based methods.

riboaligner first calls ribotyper to determine the best matching model for each sequence using special command-line options. The --minusfail, --scfail and --covfail options are used to specify that sequences with unexpected features of MinusStrand, LowScore and LowCoverage will fail. Additionally, the --inaccept $<f>$ option is used to specify that the names of the desired models to use are in file $<f>$; only sequences that match best to one of these models is eligible to pass. The default set of acceptable models is SSU.archaea and SSU.bacteria by default. All sequences that score best to one of the acceptable models are aligned to that model using the cmalign program of Infernal which takes into account both sequence and secondary structure conservation. The alignment is then parsed to determine the length classification of each sequence based on the alignment. There are 13 possible length classes which are defined based on whether the alignment of each sequence extends to or past the first and final model reference position as well as how many insertions and deletions occur in the first and final ten model reference positions. More information on these classes can be found in the Ribovore documentation.

Only sequences that pass ribotyper will be aligned by riboaligner, and the per-sequence ribotyper pass/fail designation is not changed by riboaligner. The riboaligner summary output file is identical to the ribotyper output summary file with additional per-sequence information on the length class, start and stop model reference position of each aligned sequence and number of insertions/deletions in the first and final ten model positions.

### Ribodbmaker

The ribodbmaker program is designed to create high quality datasets of rRNA sequences, which may be useful as reference datasets or blastn databases. It takes as input a set of candidate sequences and a specified rRNA model (e.g. SSU.Bacteria) and applies numerous quality control tests or filters such that only high quality sequences pass. The program performs the following steps:

1. fail sequences with too many ambiguous nucleotides (where “too many” is defined as more than the smaller of 5 and 0.5% the length of the sequence, but these thresholds can be changed by command-line options --famaxn and --famaxf)
2. fail sequences that do not have a specified species taxid in the NCBI taxonomy database
3. fail sequences that have non-weak vecscreen hits, suggesting the presence of vector contamination, as calculated by the VecScreen_plus_taxonomy software package [40]
4. fail sequences that have unexpected internal repeats as determined by comparing each sequence against itself using blastn and finding off-diagonal local alignments with an E-value of no more than 1 and length at least 20 for the plus strand and 50 for the minus strand
5. fail sequences that fail ribotyper, including matching best to a model other than the specified one, using non-default options --minusfail --lowppossc 0.5 --scfail to specify that sequences with best hits on the minus strand or with scores below 0.5 bits per nucleotide will fail
6. fail sequences that fail riboaligner, including matching best to a model other than the specified one, using non-default options --lowppossc 0.5 --tcov 0.99 to specify that sequences with scores below 0.5 bits per nucleotide or for which less than 99% of the sequence length is covered by hits will fail
7. fail sequences that do not cover a specified span of model positions (are too short)
8. fail sequences that survive all above steps but do not meet expected criteria of an *ingroup* analysis based on taxonomy and alignment identity

In step 6, riboaligner outputs multiple sequence alignments of all sequences. These alignments are used for further scrutiny of each sequence in step 7, the *ingroup* analysis step. At this stage, sequences that do not cluster (based on alignment identity) with other sequences in their taxonomic group fail. Finally, sequences that survive all stages are clustered based on alignment identity and centroids for each cluster are selected for the final set of surviving sequences.

Steps 2, 3, and 8 require access to the NCBI taxonomy database and further that each input sequence be assigned in the nucleotide database to a unique organism in the taxonomy database. This restricts the use of ribodbmaker to sequences already present in GenBank. The taxonomy criterion excludes, for example, some chimeric sequences that have been engineered and patented. Users can run ribodbmaker on other sequences, but must bypass these steps using the --skipftaxid, --skipfvecsc, and --skipingrup. The VecScreen_plus_taxonomy package is only available for Linux and so is not installed with Ribovore on Mac/OSX. Consequently, the following ribodbmaker options must be used on Mac/OSX: --skipftaxid --skipfvecsc --skipingrup --skipmstbl. In general, ribodbmaker is highly customizable via command-line option usage, and can be run using many different subsets of tests. For more information on command-line options see the ribodbmaker.md file in the Ribovore documentation subdirectory.

As described above, riboaligner calls ribotyper, so ribotyper is actually called twice by ribodbmaker, once in step 5 and once in step 6. In the riboaligner step, ribotyper is called with options that differentiate its usage from step 5, making the criteria for passing more strict in several ways. The --difffail and --multfail options are used to specify that sequences with unexpected features of LowScoreDifference and VeryLowScoreDifference will fail. Additionally, a CM is used instead of a profile HMM for the second stage (ribotyper --2slow option) and any sequence for which less than 99% of the nucleotides are covered by a hit in the second stage will fail (--tcov 0.99 option). Finally, the --scfail option, which is used in the ribotyper call in step 5, is not used in step 6.

### Ribovore reference model library and blastn databases

The Ribovore package includes 18 sequence- and structure-based alignments and corresponding CMs, listed in Table 3. The alignment and CM files are included when Ribovore is installed and are also available on GitHub. Seven of the 18 alignments are from Rfam, and the other 11 were created during development of the package. rRNA_sensor includes two blastn databases: one of 1267 bacterial and archaeal 16S SSU rRNA sequences created by clustering and filtering the blastn database already in use at GenBank in 2017 when development of the script began, and one of 1091 eukaryotic 18S SSU rRNA sequences created by filtering a sequence dataset generated by ribodbmaker.

All 18 of the Ribovore model alignments are the end products of a multi-step model refinement procedure using the valuable secondary structure data available from CRW [22] and sequences from GenBank. For each gene and taxonomic group (e.g. SSU_rRNA_eukarya), an initial alignment with consensus secondary structure was created based on combining alignments and individual sequence secondary structure predictions from CRW as described in [20, 41], and used to build a CM using the Infernal program cmbuild. That CM was then calibrated for database sequence search using cmcalibrate and searched against all currently available rRNA sequences in GenBank. The resulting high-scoring hits were then filtered for redundancy and manually examined and surviving sequences were realigned to the model to create a new alignment. In some cases the consensus secondary structure was modified slightly based on the new alignment. Some models were further refined by additional iterations of building, searching, and realigning.

Eight of the 18 Ribovore models are SSU models with fewer than 10 sequences in the training alignment (Table 3). These are for taxonomic groups with relatively few known example sequences for which the consensus secondary structure is distinct but not as well understood as for other groups, like 16S SSU rRNA. Six of these eight are non-metazoan mitochondrial models, one is a chloroplast model for the *Pilostyles* plant genus, and one is for apicoplasts. These eight models are less mature than the other ten models, but they are included in the package for completeness and we plan to improve them in future versions. Currently, users should be cautious when interpreting results that involve any of these eight models.

From each of the 18 Ribovore model alignments, two separate CMs were constructed using different command-line parameters to the cmbuild program of Infernal. One model was built using cmbuild’s default entropy weighting feature that controls the average entropy per model position [14, 20], and one was built using the cmbuild --enone option, which turns off entropy weighting. The non-entropy weighted models, which perform better at sequence classification in our internal testing (results not shown), are used by ribotyper, and the entropy weighted models are used by riboaligner for sequence alignment because they are slightly more accurate at getting alignment endpoints correct based on our own internal test results (not shown).

### Timing measurements

Timing measurements of rRNA_sensor, ribotyper, riboaligner, and ribodbmaker were done primarily on an Intel(R) Xeon(R) Gold 5118 CPU @ 2.30GHz with 48 cores and running the CentOS7.8.2003 version of Linux. We used one thread except for tests of rRNA_sensor that measured the effect on wall-clock time of increasing the number of threads. For the runs of ribodbmaker, we used the NCBI compute farm to parallelize some time intensive steps. The reason for using the compute farm only for the ribodbmaker tests is that ribodbmaker is intended primarily for curation of databases at NCBI, while the other modules are intended to be used both by submitters around the world and GenBank indexers at NCBI.

## Results and discussion

Ribovore is used directly or indirectly by NCBI and GenBank in various ways: as part of its submission pipelines for rRNA sequences, through the BLAST Web server

(https://blast.ncbi.nlm.nih.gov/Blast.cgi?PROGRAM=blastn&PAGE_TYPE= BlastSearch&LINK_LOC=blasthome)

and by facilitating the validation of sequences from type material to be incorporated into new records in the RefSeq database (https://www.ncbi.nlm.nih.gov/bioproject/224725). We detail each of these uses below and then compare the capability of Ribovore for fungal rRNA sequence validation to related projects.

### rRNA sequence submission checking

Submitters of rRNA sequences to GenBank who use the NCBI Submission Portal can choose between 12 different subtypes, listed in Table 8. For most submission subtypes, the sequences are analyzed via a blastn-based pipeline by comparing each submitted sequence against a blastn database for the specific submission subtype. Three of these submission subtypes (ITS1 and ITS2 and 16S-23S IGS) are for non-rRNA sequences. For four of the remaining nine subtypes, the blastn database currently used was created with the help of ribodbmaker, as discussed more below. The ribosensor program is used instead of the blastn pipeline to analyze 16S prokaryotic SSU rRNA submissions of 2500 or more sequences for which the submitter chooses the attribute *uncultured* to describe the sequences.

**Table 8:** NCBI rRNA and ITS sequence submission types and attributes

Submission type	Submission subtype	Validation method	Percentage of accepted submissions	Percentage of accepted sequences
Prokaryotic rRNA/IGS	SSU rRNA only (16S) ≥ 2500 seqs	ribosensor	0.648%	99.604%
	SSU rRNA only (16S) < 2500 seqs	blastn(ribo)	43.934%	0.1252%
	LSU rRNA only (23S)	blastn(ribo)	0.754%	0.0027%
	intergenic spacer (16S-23S IGS)	blastn	(sum of 2 rows)	
Eukaryotic Nuclear rRNA/ITS	Contains rRNA-ITS region	blastn	54.663%	0.2682%
	SSU rRNA only (18S)	blastn(ribo)	(sum of 8 eukaryotic rows)	
	LSU rRNA only (28S)	blastn(ribo)		
	ITS1 only	blastn		
	ITS2 only	blastn		
Eukaryotic Organellar rRNA	Mitochondrial SSU rRNA (12S)	blastn		
	Mitochondrial LSU rRNA (16S)	blastn		
	Chloroplast SSU rRNA (16S)	blastn		
	Chloroplast LSU rRNA (23S)	blastn		

The ’submission type’ column indicates the three possible rRNA sequence related options for GenBank submissions available at https://submit.ncbi.nlm.nih.gov/subs/genbank/. There is not an intergenic spacer type of eukaryotes at this time. The ’submission subtype’ column indicates the more specific possible sequence types a submitter can select after choosing one of the options in the first column. The ’validation method’ column indicates whether incoming sequences are processed with ribosensor, blastn using a database constructed with ribodbmaker (’blast(ribo)’) or blastn using either the general non-redundant (nr) database or a database constructed by other means (’blastn’). The ’percentage of accepted submissions’ and ’percentage of accepted sequences’ reflect rRNA/IGS/ITS submissions published between Jan 1, 2020 and May 31, 2020. Note that the percentages for rows 3 and 4 are summed and reported in row 3, and for rows 5 through 12 (all eukaryotic submission types) are summed and reported in in row 5. Counts pertain only to submissions that advanced through enough preliminary checks to be assigned an internal submission code

For ribosensor, the default parameters are used to determine if sequences should pass or fail as discussed in the Implementation section. For blastn, sequences are evaluated based on the average percentage identity, the average percentage query coverage, and the percentage of gaps in the alignments for the top target sequences. Additionally, using a blastn-based method that predates and inspired rRNA_sensor, sequences that are suspected to be misassembled or incorrectly labelled taxonomically fail. Specifically, the query sequence is tested with blastn against the 16S SSU rRNA database described below and the matches are ranked in increasing order of E-value. A sequence passes the misassembly test if and only if the best matches each have exactly one local alignment. The taxonomy tests are based on a comparison of the proposed taxonomy from the submitter and the taxonomic information of the top matches, taking into account variant spellings and synonyms in NCBI Taxonomy. The exact thresholds for these pre-Ribovore, blastn-related comparisons vary according to the type of submission and are outside the scope of this paper.

For both the blastn and ribosensor pipelines submissions in which all sequences pass and which have the required metadata are automatically deposited into GenBank. All other submissions fail and are either sent back to the submitter with automated error reports or manually examined further by GenBank indexers, depending on the specific reason for the failure.

A key objective of distributing the Ribovore software is to permit submitters to do on their own computers similar checks to those done by the GenBank submission pipeline. In 2018, we began using earlier versions of ribosensor to analyze large-scale 16S prokaryotic SSU rRNA submissions. This remains the only submission type for which ribosensor is employed in an automated way, although we plan to expand to additional genes and taxonomic domains in the future. Parts of Ribovore are also used manually by GenBank indexers to evaluate some submissions.

The most common type of rRNA submission by far is 16S prokaryotic SSU rRNA (Table 8). Between July 1, 2018 and May 31, 2020, 33,388 submissions of 16S SSU rRNAs with less than 2500 sequences (or for which the submitter indicated the sequences were from cultured organisms), were handled by the blastn pipeline. The total number of sequences in these submissions was 240,112, for an average of 7.2 sequences per submission. In the same time interval, ribosensor processed 242 16S SSU rRNA submissions comprising 49,868,017 sequences for an average of 206,066.2 sequences per submission. In the first six months of 2020, ribosensor processed more than 99.6% of the sequences deposited in GenBank via any of the the rRNA or ITS submission pipelines (Table 8).

### Construction and usage of rRNA databases for blastn

Four of the ten rRNA blastn databases used for submission checking were created by ribodbmaker as indicated in Table 8. An additional three blastn databases, available in the web server version of BLAST are described below. All blastn databases we mention can be retrieved for local use from the directory https://ftp.ncbi.nlm.nih.gov/blast/db/ The databases are (re)generated semi-automatically by extracting large sets of plausible sequences from Entrez and providing them as input to ribodbmaker. The ribodbmaker program is run so that the tests for ambiguous nucleotides, specified species, vector contamination, self-repeats, ribotyper, riboaligner, and model span are all executed. However, the databases are allowed to contain more than one sequence per taxid and the ingroup analysis is skipped (--skipingrup option). Only sequences that pass ribodbmaker tests are eligible to be in the blastn databases. To keep the 16S prokaryotic SSU, eukaryotic SSU, and eukaryotic LSU databases to a reasonable size, the sequences that pass ribodbmaker are clustered with UCLUST [37] at a threshold of 97% identity and all other parameters at default values. The clustering stage of ribodbmaker is not used for this purpose and is skipped by using the --skipclustr option.

The 16S prokaryotic SSU rRNA BLAST database is generated starting from all sequences in the GenBank nucleotide database that match NCBI BioProject IDs PRJNA33175 or PRJNA33317 using the eutils query in the first row of Table 9. PRJNA33175 has the title “Bacterial 16S Ribosomal RNA RefSeq Targeted Loci Project”. PRJNA33317 has the title “Archaeal 16S Ribosomal RNA RefSeq Targeted Loci Project”. This formal query is supplemented by manual searches of the journal *International Journal of Systematics and Evolutionary Biology* (https://www.microbiologyresearch.org/content/journal/ijsem), where many new bacterial species are announced and peer-reviewed, along with their 16S SSU rRNA sequences. Among the databases described here, the 16S SSU rRNA database is the only one restricted to sequences from “type material” that have been more stringently vetted before curation for RefSeq. The fungal RefSeq records described in a later subsection are also restricted to be from “type material”.

**Table 9:** Queries used in command-line eutils to collect input datasets for ribodbmaker

Gene	**Eutils** Query
Archaeal and bacterial SSU rRNA	PRJNA33175[BioProject] OR PRJNA33317[BioProject]
Bacterial LSU rRNA	Bacteria [orgn] AND (23S [ti] OR large subunit ribosomal RNA [ti])
	NOT uncultured [orgn] NOT 23S rRNA methyltransferase [ti] NOT srcdb pdb [prop]
	NOT srcdb PAT [prop] NOT WGS [filter] NOT mRNA [filter] [ti]
	NOT RefSeq [filter] NOT mRNA NOT “mitochondrion”[Filter] NOT TLS [ti]
Archaeal LSU rRNA	Archaea[orgn] AND (23S [ti] OR large subunit ribosomal RNA [ti])
	NOT uncultured [orgn] NOT 23S rRNA methyltransferase[ti] NOT srcdb pdb [prop]
	NOT srcdb PAT [prop] NOT WGS [filter] NOT mRNA [filter] [ti]
	NOT RefSeq [filter] NOT mRNA NOT “mitochondrion”[Filter] NOT TLS [ti]
Eukaryotic SSU rRNA	eukaryota[orgn] (18S [ti] OR small subunit ribosomal RNA [ti])
	NOT WGS [filter] NOT mRNA [filter] NOT “mitochondrion”SSU rRNA [Filter]
	NOT plastid [filter] NOT chloroplast [filter] NOT plastid [ti]
	NOT chloroplast [ti] NOT mitochondrial [ti] NOT RefSeq [filter]
	NOT (5.8S [ti] OR internal [ti]) NOT 28S [ti] NOT WGS NOT mRNA
	NOT “mitochondrion”[Filter] NOT TLS [ti] NOT srcdb pdb[prop]
Eukaryotic LSU rRNA	Eukaryota[orgn] AND (25S [ti] OR 26S [ti] OR 28S [ti] OR large subunit
	Ribosomal RNA [ti]) NOT WGS [filter] NOT mRNA [filter] NOT “mitochondrion”
	[Filter] NOT plastid [filter] NOT chloroplast [filter] NOT plastid [ti]
	NOT chloroplast [ti] NOT mitochondrial [ti] NOT RefSeq [filter] NOT
	(5.8S [ti] OR internal [ti]) NOT TLS [ti] NOT partial cds [ti] NOT
	Chain [ti] NOT 18S [ti] NOT srcdb pdb [prop]
Microsporidia SSU rRNA	Microsporidia [orgn] AND (18S [ti] OR small subunit ribosomal RNA [ti])
	NOT WGS [filter] NOT mRNA [filter] NOT “mitochondrion”[Filter]
	NOT plastid [filter] NOT chloroplast [filter] NOT plastid [ti]
	NOT chloroplast [ti] NOT mitochondrial [ti] NOT RefSeq [filter]
	NOT (5.8S [ti] OR internal [ti]) NOT 28S [ti] NOT WGS NOT mRNA
	NOT “mitochondrion”[Filter] NOT TLS [ti] NOT srcdb pdb[prop]
Microsporidia LSU rRNA	Microsporidia [orgn] AND (25S [ti] OR 26S [ti] OR 28S [ti] OR large subunit
	Ribosomal RNA [ti]) NOT WGS [filter] NOT mRNA [filter] NOT “mitochondrion”
	[Filter] NOT plastid [filter] NOT chloroplast [filter] NOT plastid [ti]
	NOT chloroplast [ti] NOT mitochondrial [ti] NOT RefSeq [filter]
	NOT (5.8S [ti] OR internal [ti]) NOT TLS [ti] NOT partial cds [ti]
	NOT Chain [ti] NOT 18S [ti] NOT srcdb pdb [prop]
Fungal SSU rRNA RefSeq records	Fungi [orgn] AND 225:10000 [slen] AND sequence from type [filter]
	AND (18S [ti] OR small subunit ribosomal RNA [ti]) NOT WGS [filter]
	NOT mRNA [filter] NOT mitochondrion [Filter] NOT plastid [filter]
	NOT chloroplast [filter] NOT mitochondrial [ti] NOT (5.8S [ti] OR internal [ti])
	NOT 28S [ti] NOT 26S [ti] NOT 25S [ti] NOT 5S [ti] NOT 23S [ti] NOT 37S [ti]
	NOT WGS NOT mRNA NOT RefSeq [filter] NOT TLS [ti]
Fungal LSU rRNA RefSeq records	Fungi [orgn] AND 425:10000[slen] AND sequence from type[filter]
	AND (25S[ti] OR 26S[ti] OR 28S [ti] OR large subunit ribosomal RNA[ti])
	NOT WGS[filter] NOT mRNA [filter] NOT “mitochondrion”[Filter]
	NOT plastid [filter] NOT chloroplast [filter] NOT mitochondrial [ti]
	NOT (5.8S [ti] OR internal [ti]) NOT TLS [ti] NOT partial cds [ti]
	NOT Chain [ti] NOT 18S [ti] NOT srcdb pdb [prop] NOT RefSeq [filter]

Table 9 also lists the eutils queries to the nucleotide database that are used for 23S prokaryotic LSU rRNA, eukaryotic SSU rRNA, eukaryotic LSU rRNA, *Microsporidia* SSU rRNA, and *Microsporidia* LSU rRNA. When we seek sequences that are likely to be complete, not larger genome pieces, and not partial, we add a constraint on the length with an extra term such as 1500:18000[slen] for eukaryotic SSU rRNA. The main attribute that distinguishes *Microsporidia* is that the lower bound on slen for complete sequences is set about 300–500 nucleotides lower as explained in Background. To find possibly partial LSU sequences that are long enough to cover the variable regions, we add the condition 425:1000[slen]. These queries rely on standardized nomenclature and structure of the *definition line* of GenBank sequence records which contain information about the source organism, feature content, completeness and location. Since 2016, these definition lines have been constructed formulaically during the processing of submissions. For example, the sequence MT981756.1 has the title: “Staphylococcus epidermidis strain RA13 16S ribosomal RNA gene, partial sequence”, and the sequence MN158348.1 has the title “Tetrahymena rostrata strain TRAUS 18S ribosomal RNA gene, internal transcribed spacer 1, 5.8S ribosomal RNA gene, internal transcribed spacer 2, and 28S ribosomal RNA gene, complete sequence”.

### Creation of fungal rRNA RefSeq entries using ribodbmaker

NCBI’s RefSeq project seeks to create a representative, non-redundant set of annotated genomes, transcripts, proteins and nucleotide records including rRNA sequences [36]. Since 2018, ribodbmaker has been used to screen the set of fungal 18S SSU rRNA and 26S LSU rRNA sequences. Table 9 lists the queries used to identify candidates to be new fungal SSU and LSU rRNA RefSeq records.

Studies that target fungal rRNAs frequently attempt to obtain SSU rRNA sequences that span most of the V4 and part of the V5 variable regions, or LSU rRNA sequences that span the D1 and D2 variable regions as these have been shown to be phylogenetically informative [42, 43]. These regions correspond to Rfam RF01960 model positions 604 to 1070 (SSU) and RF02543 model positions 124 to 627 (LSU). Correspondingly, ribodbmaker is run with command-line options (--fmlpos and --fmrpos) that enforce that only sequences that span these model coordinates can pass. The following ribodbmaker options are used for SSU: --fione --fmnogap --fmlpos 604 --fmrpos 1070 -f --model SSU.Eukarya --skipclustr, and for LSU: --fione --fmnogap --fmlpos 124 --fmrpos 627 -f --model LSU.Eukarya --skipclustr.

### NCBI BLAST webpage rRNA target databases

For many years, NCBI has been offering searches of nucleotide and protein databases with various modules of BLAST [44] through the NCBI BLAST webpage. Most commonly, searches of nucleotide queries use a comprehensive “nonredundant (nr)” database of nucleotide sequences or databases of whole genomes. A disproportionate number of queries are rRNA sequences. When blastn users know that their queries are of these special types, searching smaller targeted databases that exclude sequences unexpected to have a significant match to the query reduces running time and leads to more focused results. The BLAST webpage now allows users to select from three ribodbmaker-derived rRNA target databases, listed in Table 10. The 16S SSU rRNA database is identical to the one used by the blastn submission pipeline. The other two are specific to *Fungi*, due to the popularity of the analysis of rRNA sequences for studies of that kingdom. The fungal SSU and fungal LSU BLAST databases are effectively equivalent to the sets of curated RefSeq records described below. The availability of these databases was announced in late 2019, and the number of blastn runs and unique blastn visitors who selected each database during the seven-month period November 12, 2019–June 22, 2020 are reported in Table 10. The usage suggests that there is sufficient user demand to justify the curation effort. As of November 24, 2020, there are 2836 fungal SSU RefSeq records and 7573 fungal LSU RefSeq records, almost all of which were curated with Ribovore.

**Table 10:** Web blastn usage of specialized rRNA databases that are curated using Ribovore to decide which sequences are valid

Database	Runs	Runs/day	Visits	Visits/day
16S rRNA sequences (SSU) from Bacteria and Archaea	355,479	1921.5	92,638	500.7
18S rRNA sequences (SSU) from Fungi and reference type material	5724	30.9	2876	15.5
28S rRNA sequences (LSU) from Fungi and reference type material	4535	24.5	1861	10.1

Usage was measured during November 12, 2019–June 22, 2020

### Comparison to curated sets of fungal rRNA sequences from Silva

We compared iteratively our ribodbmaker approach to curating fungal RefSeq records with other curatorial efforts that are part of the Silva project [27]. The purposes of this comparison were:

1. to identify new candidate RefSeq records and possible weakness in our procedures for choosing RefSeq records,
2. to test whether Ribovore works on curated data sets and to correct errors associated with some sequences in NCBI databases, such as misleading definition lines,
3. to characterize what proportion of sequences curated by others pass the Ribovore criteria and why sequences fail.

As noted above, fungal LSU sequences submitted since 2016 or 2017 should have definition lines that match this query. In the course of doing the SilvaParc LSU tests described below, we corrected the definition lines of 132 older sequences that are fungal LSU and passed all ribodbmaker tests, but did not match the above eutils query. The current fungal RefSeq SSU and LSU sequences can be obtained with the queries “PRJNA39195[BioProject]” and “PRJNA51803[BioProject]”, respectively or both from the ftp site: https://ftp.ncbi.nlm.nih.gov/refseq/TargetedLoci/Fungi/.

For our comparison of fungal sequences, we used a curated set of 8770 SSU sequences from Silva [45], a set of 1461 SSU sequences from Silva in the phylum *Microsporidia*, a set of 2993 high-quality LSU reference sequences from Silva, and a much larger set of 394,247 sequences from Silva called Parc [26, 46]. We denote these four sets as *Yarza*, *SilvaMicrosporidia*, *SilvaRef*, and *SilvaParc*, respectively. To set up the SilvaMicrosporidia set, we downloaded the FASTA files for all of SilvaParc SSU and extracted 1,468 sequences labeled as being from the phylum *Microsporidia*; of these, 1461 sequences were in GenBank with sufficient taxonomy information to be considered for ribodbmaker. SilvaParc contains fewer than 50 *Microsporidia* LSU sequences, supporting our previous assertion that the SSU has been much more studied than the LSU in *Microsporidia*.

Similarly, to set up the SilvaRef and SilvaParc sets, we downloaded FASTA files for all SilvaRef LSU sequences for all taxa in version 132 on July 23, 2020 and all LSU Parc sequences in version 132 on August 1, 2020. We filtered for all sequences that had the token “Fungi” in the definition line. A small number of sequences had to be dropped subsequently because 1) they are not from the kingdom *Fungi* (e.g., they may be from a pathogen of a fungus) 2) they were absent from the nuccore database of GenBank either due to being “unverified” or from certain types of patents or 3) due to phylogenetic discrepancies (cf. [47]) that we subsequently fixed as part of the first objective listed above. In all data sets, we retrieved the most recent version of all GenBank accessions, which differs from the curated version for a very small number of sequences since version 132 of Silva is recent. In our analysis of the SilvaParc set, 96 non-fungal sequences were inadvertently included in the analysis, and excluded only while checking the results. The results of the three comparisons are shown in Table 11.

**Table 11:** Summary of ribodbmaker pass/fail outcomes for Yarza(SSU), SilvaMicrosporidia(SSU), SilvaRef(LSU), and SilvaParc(LSU) datasets

Dataset/test	Yarza pass/fail	SilvaMicrosporidia pass/fail	SilvaRef pass/fail	SilvaParc pass/fail
Ambiguous nucleotides	8493/277	1435/26	2928/65	385607/8640
Specified species	7543/1227	774/687	2089/904	204923/189324
Vector contamination	8735/35	460/1	2989/4	393938/305
Self-repeats	8635/135	1418/43	2929/64	390305/3942
Ribotyper	8085/685	1140/321	2925/68	145450/248797
Riboaligner	7720/1050	998/463	1583/1410	135252/258995
Length in range?	7617/103	957/41	1187/396	132984/2268
Expected span?	7612/5	669/288	990/197	103016/29968
All 1-sequence tests?	6359/2411	405/1056	759/2234	70127/324120
Ingroup analysis (many)	5158/1201	288/117	649/110	56606/13521
Ingroup analysis (1)	3246/3113	136/269	478/281	7129/52998

All tests except the ingroup analysis depend only on the sequence being tested. The four tests for ambiguous nucleotides, specified species, vector contamination, and self-repeats are done on all sequences, so sequences may fail more than one test. Only sequences that pass the ribotyper test are eligible as input to riboaligner. Only sequences that pass the riboaligner test are eligible to be tested for length and alignment span. Only sequences that pass all 1-sequence tests are eligle for ingroup analysis. The ingroup analysis can be done allowing many sequences from the same taxon to pass or limiting to 1 the number of sequences that pass from each taxon (argument --fione). The many option is a more meaningful test; we show the 1 option just for comparison. Data and instructions for reproducing these comparisons are available at https://github.com/nawrockie/ribovore-paper-2021-supplementary-material

The main steps in these tests consisted of:

1. Download and uncompress a FASTA file of source sequences from the supplementary information of [45] or from the Silva FTP retrieval site, which we denote File1.fa.
2. As explained in the Ribovore documentation, retrieve and condense the current version of NCBI’s taxonomy tree. An important and subtle column is the boolean (0/1) specified species column for each taxon; a 1 in this column for the row of taxon *t* means that according to NCBI’s Taxonomy Group the taxon name is valid and currently peferred; a 1 in this column is a necessary condition for a sequence from taxon *t* to be eligible to be in the rRNA databases or to be a RefSeq. Call the resulting file taxonomy.txt.
3. (For tests of Silva data only) Extract the definition lines for sequence identifiers of interest with the command: grep Fungi File1.fa | grep -v Bacteria or grep Microsporidia File1.fa | grep -v Bacteria, redirecting the output to an intermediate file. The second command in the pipe removes most sequences from fungal pathogens that are not actually from the kingdom of Fungi. For the Fungal SSU set, all sequences are of interest, so the simpler command grep “>” File1.fa extracts the definition lines.
4. Extract GenBank accessions without the versions from the definition lines at step 3. Versions are removed because for some sequences, the version in Silva has been superseded in GenBank with a newer version.
5. Use the NCBI package eutils [48] to retrieve from the nucleotide database of GenBank all currently live accessions from the accession sets derived at the previous step. Some sequences get dropped at this step because they are no longer live. Call the FASTA file at this step File2.fa.
6. Use the NCBI standalone tool srcchk (available at: ftp://ftp.ncbi.nih.gov/toolbox/ncbi_tools/converters/by_program/srcchk/) to check which sequences in File2.fa have a valid and fully consistent taxonomy entry. Remove sequences that do not get a normal result from srcchk because they will cause Ribovore to halt. A small number (well below 1%) of sequences get removed at this step either because they are engineered sequences from patents or because there are transient inconsistencies between the NCBI taxonomy tree and the organism values in the GenBank nucleotide records. Call the resulting file File3.fa.
7. Run ribodbmaker --taxin taxonomy.txt --skipclustr --model <model-value> --fmlpos <left-boundary> --fmrpos <right-boundary> --fmnogap --fione --pidmax 71000 --indiffseqtax -f -p <output directory>. The value of <model-value> was either SSU.Eukarya, SSU.Microsporidia, or LSU.Eukarya, depending on the test being done. The values of <right-boundary> and <left-boundary> are set in a model-specific manner according to the recommended values in the Ribovore documentation (ribodbmaker.md file in documentation subdirectory). The 71000 is an upper bound on the number of sequences input in our various tests. Version 0.40 of Ribovore was used.

For the large test of SilvaParc, we ran the last ribodbmaker step separately on various subsets of File3.fa, then collected all sequences that passed all sequence-specific tests, and did a final run of ribodbmaker to include the ingroup analysis. This split into multiple runs achieves better parallelism and throughput for large versions of File3.fa because the ingroup analysis is the only step in which the results for any single sequence depend on which other sequences are included in the input. The numbers of eligible sequences reported in Table 11 are those included in File3.fa. The results are slightly sensitive to changes in the NCBI taxonomy tree, which is updated daily. For the Yarza tests, we used the NCBI taxonomy tree as of August 1, 2020 and for the Silva tests we used the tree as of September 22, 2020.

It was not our intent to compare sets of “passing” sequences because the criteria for fungal RefSeq records are deliberately more stringent than for inclusion in Silva. Most notable are the two taxonomic tests: 1) that each sequence should come from a specified species and 2) that in selecting sequences for RefSeq, we may choose to keep only one sequence per species taxid, as specified by the command-line option --fione, to avoid redundancy.

Indeed, the analysis of fungal sequences from Silva yielded some new fungal RefSeq records; specifically, we added 4 SSU sequences from the Yarza set, and 10 LSU sequences from the SilvaRef and SilvaParc data sets. However, the tests results also show some possible improvements in the Silva curation. It appears that a small number of Silva sequences have vector contamination and more than 1% may be misassembled as indicated by self-repeats, which are not expected in fungal SSU and LSU rRNA genes (see Methods, subsection ribodbmaker for the self-repeat criteria). It appears that the Yarza SSU data set was carefully curated for sequences to be full-length and not too long, but in the SilvaRef and SilvaParc data sets more than 20% of sequences have either a length that is out of the range of typical eukaryotic LSU sequences or do not span the range [124,627] that includes the D1/D2 regions typically covered for species differentitation. Thus, it appears that the Silva resource curation could arguably be improved by checking sequence ends, so as to trim long sequences, remove short sequences, and remove sequences that are unlikely to be full LSU sequences. The sequences could be too long either because they were not trimmed to the LSU boundary or possibly because they contain introns. In the SilvaMicrosporidia test, we tested for the presence of the most conserved V4 and V5 regions only with a permissive expected span of [380,800].

### Partial comparison to RNAmmer

To our knowledge, there is no other software that solves the rRNA validation problem as we have formulated it for GenBank submissions. One widely used software package that solves a related problem is RNAmmer [28]. The problem that RNAmmer solves is to find likely rRNA sequences within larger sequences by using an old version of HMMER (v2.3.2) to compare against one of six profile HMMs. To accelerate searches, RNAmmer first utilizes a small *spotter* profile HMM that models only the most conserved 75 consecutive positions of the overall rRNA alignment to detect rRNA regions, padding those regions with extra sequence on each end, and then using a profile of the full rRNA to determine gene boundaries within those padded regions. So far as we could determine, RNAmmer does not work with the up-to-date HMMER version 3 nor with arbitratry profile HMMs or CMs.

Nevertheless, one can provide as input to RNAmmer FASTA files of putative rRNA sequences, pretending that they were larger contigs. The module of Ribovore that is closest in purpose to this usage of RNAmmer is ribotyper. To allow comparison between RNAmmer and ribotyper, one can define that a sequence *passes* RNAmmer if RNAmmer produces in the output at least one HMMER-based prediction for that sequence using the intended rRNA model (e.g., eukaryotic SSU for the Yarza set) and *fails* if there are zero such predictions. This comparison is unfair to RNAmmer because it does not use the predicted intervals, which are the most useful part of the output when RNAmmer is used with large contigs as inputs.

We compared the performance of ribotyper versus RNAmmer on the Yarza SSU set and the SilvaRef LSU set. We used the ribotyper results obtained from the ribodbmaker tests described above and summarized in Table 11. Among the 8770 SSU sequences in the Yarza set: 7,999 passed both ribotyper and RNAmmer, 23 failed both ribotyper and RNAmmer, 665 passed RNAmmer and failed ribotyper, and 86 passed ribotyper and failed RNAmmer. Among the set of 665, 557 sequences include “internal transcribed spacer” or “ITS” in the definition line, and 69 of the other 108 have lengths above 5000 nt, indicating that all but at least 39 of the sequences likely include sequence outside the SSU rRNA sequence (which is rarely more than 3Kb) and so are expected to fail ribotyper. Of the 86 sequences that passed ribotyper and failed RNAmmer, 46 of them would pass RNAmmer if the E-value and bit score thresholds for the spotter HMM which are hard-coded at 1E-5 and 0 were changed to 1 and -100 in the rnammer Perl script, indicating that these sequences do not match well to the spotter profile HMM used for eukaryotic SSU rRNA.

Among the 2993 LSU sequences in the SilvaRef set: 2481 passed both RNAmmer and ribotyper, 21 failed both RNAmmer and ribotyper, 47 passed RNAmmer and failed ribotyper, and 444 passed ribotyper and failed RNAmmer. Among the 47 sequences that passed RNAmmer and failed ribotyper, 44/47 can be explained because they have one of three errors that ribotyper looks for and would not necessarily lead RNAmmer to have no output matches: R_DuplicateRegion (24 sequences), R_BothStrands (7 sequences), R_MultipleFamilies (13 sequences). Many of the 47 sequences are described on the definition lines as a “shotgun assembly”; accordingly, the R_DuplicateRegion and R_BothStrands errors indicate two different errors that occur commonly in assembling nucleotide sequences into contigs. Some of the sequences have both SSU and LSU in the definition lines and if accurate, that should lead to an R_MultipleFamilies error. These 13 sequences that match both genes could have been trimmed before inclusion in the SilvaRef LSU set. In principle, one could detect the presence of an SSU match and an LSU match in the same sequence with RNAmmer, but one would have to add error rules to RNAmmer to decide when the occurrence of matches to both SSU and LSU models is an error. That need for error semantics exemplifies how ribotyper differs in functionality from RNAmmer. Of the 444 sequences that passed ribotyper and failed RNAmmer, 433 of them would pass RNAmmer if, as for the Yarza set, the E-value and bit score thresholds for the spotter HMMs were changed to 1 and -100 respectively, indicating that these sequences do not match well to the eukaryotic LSU rRNA spotter profile HMM. Data and instructions for reproducing these comparisons are available at https://github.com/nawrockie/ribovore-paper-2021-supplementary-material.

In general, there are a large number of discrepant outcomes and neither RNAmmer nor ribotyper is consistently more restrictive than the other. We infer merely that the two pieces of software solve different problems and there is not a straightforward way to modify RNAmmer to solve the problem of checking rRNA submissions to GenBank. This helps to justify why we developed the new software Ribovore. As explained above, Ribovore also has additional modules, such as ribodbmaker, that are even less comparable to RNAmmer and solve other problems in rRNA sequence validation and curation.

### Limitations and future directions

Ribovore includes 18 profile models (Table 3), only two of which are used for automated submission checking (bacterial SSU rRNA and archaeal SSU rRNA), and seven of which (the first seven rows in Table 3) have been used in the context of ribodbmaker to generate one or more blastn databases or RefSeq records. Eight of the remaining models were created from alignments of fewer than 10 sequences, and need to be improved by adding more sequences. However, some of the models, especially those based on Rfam alignments such as eukaryotic SSU and LSU rRNA, could in principle be used for submission checking by ribosensor and we plan to investigate those possibilities based on empirical testing in the future. Beyond the existing models, more models are needed for other rRNA genes and taxonomic domains, such as mitochondrial LSU rRNA, *Microsporidia* LSU rRNA, eukaryotic 5.8S rRNA and 5S rRNA. Rfam includes alignments for some of these (e.g. 5.8S and 5S rRNA) and future versions of Ribovore could include models based on those, but manual curation effort will be required to create others.

One limitation of Ribovore is that there are many parameters and the user may need to choose the settings carefully for each distinct purpose. For example, the usage of ribodbmaker should be tuned for each gene and taxonomic domain, as we have reported here for fungal SSU and LSU rRNA to require the commonly targeted regions of those respective genes to be present in the sequences. Another limitation is that we do not model introns, simply expecting any introns to be unaligned in the ribotyper and riboaligner analysis. Additionally, minimum criteria (e.g. minimum score and coverage values) for passing sequences in the ribotyper, ribosensor and riboaligner tools should be set based on empirical testing, and the default values for those programs are currently tailored to prokaryotic SSU rRNA based on our internal testing. Expansion to other genes and taxonomic domains will require additional testing of those values.

For some applications, the running time of Ribovore programs can be a significant limitation. Profile-based CM or profile HMM methods that compare a few profiles (in this case, at most 18) to each input sequence can be more efficient than single-sequence based methods like blastn which typically compare many database sequences (in this case, more than 1000) to each input sequence, but of course this depends on the relative speed of each profile to sequence and sequence to sequence comparison. CM methods that score both sequence and secondary structure conservation are computationally complex, requiring time $O(N^4)$ for a sequence of length N without heuristic filtering or banded dynamic programming approaches [49]. Even with heuristic-based acceleration strategies, alignment of a single full length LSU rRNA sequence typically takes several seconds on a single CPU. For this reason, ribotyper and ribosensor, which are intended to handle sequence submissions of up to millions of sequences do not, by default, compute an alignment using the CM, but rather use only more efficient profile HMM algorithms. The riboaligner and ribodbmaker programs, however, do compute alignments using CMs and so take longer per sequence, although the frequency with which these programs need to be run, at least for GenBank, is less. The ribosensor program, which runs both ribotyper and rRNA_sensor, which is blastn-based, combines both profile and single-sequence methods.

We measured the running time of rRNA_sensor by itself and ribosensor on 1000 16S sequences using 1, 2, 4, 8, and 16 processors. The rRNA_sensor program took 199s, 104s, 54s, 29s, and 17s, respectively; ribosensor took 362s, 196s, 106s, 61s, 39s, respectively. Thus, a submission of 1 million sequences, which is on the high end, would take hours to process given 16 processors on the host computer. The programs ribotyper, ribosensor, riboaligner, and ribodbmaker all include a command-line option -p <n> that enables parallelization by splitting the input file into <n> roughly equal sized chunks and processing each chunk independently on <n> nodes on a compute cluster. However, for ribodbmaker, only the ribotyper and riboaligner steps are parallelized by splitting the input file into chunks of similar size.

While doing the comparison of curated fungal datasets, we also timed ribotyper, riboaligner, and ribodbmaker on the 8770 sequence Yarza fungal SSU set and the 2993 sequence SilvaRef fungal LSU set as described in Implementation. The ribotyper program required 40m 14s (0.275s per sequence) on the Yarza set and 16m 51s (0.338s per sequence) on the SilvaRef set. The riboaligner program took 116m 22s (0.796s per sequence) on the Yarza set and 123m 56s (2.48s per sequence) on the SilvaRef set. The ribodbmaker program took 49m 49s wall-clock time and 1191m 2s cumulative time for all processors on the Yarza set and 60m 24s wall-clock time and 980m 11s cumulative time for the SilvaRef set. In general, we conclude that these analyses are tractable for tens of thousands of sequences at a time.

## Conclusions

Our primary contribution described herein is the software package Ribovore for rRNA sequence analysis. At NCBI since July 2018, Ribovore has been used to check the quality of incoming submissions and to curate datasets of high quality sequences for RefSeq or to use as blastn databases. In the submission checking context, Ribovore has been used to check nearly 50 million 16S bacterial and archaeal SSU rRNA sequences through May 31, 2020 and millions more after that date. Ribovore has also been used manually by GenBank indexers when blastn analyses gave uncertain results for other rRNAs. A subset of the blastn databases created by Ribovore are selectable by users of the BLAST webpage as target databases, and are used in over 2000 web blastn runs per day. We also are using Ribovore internally to curate fungal RefSeq records for SSU and LSU rRNA from type material. We showed that this curation effort is complementary to the larger Silva effort, as it selects only the best sequences that pass a larger battery of tests. Furthermore, the RefSeq records are linked within Entrez to other NCBI resources including BioCollections, BioProjects, Taxonomy, and BLAST. With this formal report of how Ribovore is designed and implemented, we hope that both producers and consumers of rRNA sequence data will achieve a new understanding of how rRNA sequences are curated in GenBank, RefSeq, and associated resources.

## Availability and requirements

Project name: Ribovore

Project home page: https://github.com/ncbi/ribovore

Operating system(s): Linux, Mac/OSX

Programming language: Perl

Other requirements: BLAST+ v2.11.0, Infernal v1.1.4, Sequip v0.08, VecScreen_plus_taxonomy v0.17 (see Table 2)

License: public domain

Any restrictions to use by non-academics: none

## Data Availability

All data generated or analyzed during this study are included in this published article, its supplementary material, or NCBI’s GenBank database. Code is available on GitHub (https://github.com/ncbi/ribovore). BLAST databases are available in the directory https://ftp.ncbi.nlm.nih.gov/blast/db/. The supplementary material is available on GitHub (https://github.com/nawrockie/ribovore-paper-2021-supplementary-material) and includes instructions for reproducing the comparisons reported in the article.

## Abbreviations

NCBI: National Center for Biotechnology Information
rRNA: Ribosomal RNA
SSU rRNA: Small subunit ribosomal RNA
LSU rRNA: Large subunit ribosomal RNA
CM: Covariance model
HMM: Hidden Markov model
nt: Nucleotides
Kb: Kilobase (1000 nucleotides)

## References

[1] Woese CR, Fox GE (1977). Phylogenetic structure of the prokaryotic domain: the primary Kingdoms. Proc Natl Acad Sci USA.

[2] Pace NR, Stahl DA, Lane DJ, Olsen GJ (1985). Analyzing natural microbial populations by rRNA sequences. ASM News.

[3] Weller R, Ward DM (1989). Selective recovery of 16S rRNA sequences from natural microbial communities in the form of cDNA. Appl Environ Microbiol.

[4] Giovannoni SJ, Britschgi TB, Moyer CL, Field KG (1990). Genetic diversity in Sargasso sea bacterioplankton. Nature.

[5] Fox GE, Pechman KR, Woese CR (1977). Comparative cataloging of 16S ribosomal ribonucleic acid: molecular approach to procaryotic systematics. Int J Syst Evol Microbiol.

[6] Betzl D, Ludwig W, Schleifer KH (1990). Identification of em Lactococci and enterococci by colony hybridization with 23S rRNA-targeted oligonucleotide probes. Appl Env Microbiol..

[7] Amann RI, Ludwig W, Schleifer KH (1995). Phylogenetic identification and in situ detection of individual microbial cells without cultivation. Microbiol Rev.

[8] Begerow D, Nilsson H, Unterseher K, Maier W (2010). Current state and perspectives of fungal DNA barcoding and rapid identification procedures. Appl Microbiol Biotech.

[9] Schoch CL, Seifert KA, Huhndorf S, Robert V, Spouge JL, Levesque CA (2012). Nuclear ribosomal internal transcribed spacer (ITS) region as a universal DNA barcode marker for fungi. Proc Natl Acad Sci USA.

[10] Peterson SW, Kurtzman CP (1991). Ribosomal RNA sequence divergence among sibling species of yeasts. Syst Appl Microbiol.

[11] Pawlowski J, Audic S, Adl S, Bass D, Belbhari L, Berney C (2012). The significance of a confidence between evolutionary landmarks found in mating affinity and a DNA sequence. PLOS Biol.

[12] Zimmerman J, Hahn R, Geimenholzer B (2011). Barcoding diatoms: evaluation of the V4 subregion on the 18S rRNA gene, including new primers and protocols. Org Divers Evol.

[13] Eddy SR (1998). Profile hidden Markov models. Bioinformatics.

[14] Karplus K, Barrett C, Hughey R (1998). Hidden Markov models for detecting remote protein homologies. Bioinformatics.

[15] Eddy SR, Durbin R (1994). RNA sequence analysis using covariance models. Nucleic Acids Res.

[16] Sakakibara Y, Brown M, Underwood RC, Mian IS, Haussler D. Stochastic Context-Free Grammars for Modeling RNA. In: Hunter L, editor. Proceedings of the twenty-seventh annual hawaii international conference on system sciences: biotechnology computing. vol. V. Los Alamitos, CA: IEEE Computer Society Press; 1994. p. 284–293.

[17] Durbin R, Eddy SR, Krogh A, Mitchison GJ (1998). Biological sequence analysis: probabilistic models of proteins and nucleic acids.

[18] Freyhult EK, Bollback JP, Gardner PP (2007). Exploring genomic dark matter: a critical assessment of the performance of homology search methods on noncoding RNA. Genome Res.

[19] Kolbe DL, Eddy SR (2009). Local RNA structure alignment with incomplete sequence. Bioinformatics.

[20] Nawrocki EP, Structural RNA. Homology search and alignment using covariance models [Ph.D. Thesis]. Washington University School of Medicine; 2009.

[21] Ludwig W, Strunk O, Westram R, Richter L, Meier H (2004). ARB: a software environment for sequence data. Nucleic Acids Res.

[22] Cannone JJ, Subramanian S, Schnare MN, Collett JR, D’Souza LM, Du Y, et al. The comparative RNA web (CRW) site: an online database of comparative sequence and structure information for ribosomal, intron, and other RNAs. BMC Bioinform. 2002;3:2. 10.1186/1471-2105-3-2 PMC65690 11869452

[23] Olsen GJ, Larsen N, Woese CR (1991). The ribosomal RNA database project. Nucleic Acids Res.

[24] Cole JR, Wang Q, Fish JA, Chai B, McGarrell DM, Sun Y (2014). Ribosomal database project: data and tools for high throughput rRNA analysis. Nucleic Acids Res.

[25] DeSantis TZ, Hugenholtz P, Larsen N, Rojas M, Brodie EL, Keller K (2006). Greengenes, a chimera-checked 16S rRNA gene database and workbench compatible with ARB. Appl Environ Microbiol.

[26] Pruesse E, Quast C, Knittel K, Fuchs BM, Peplies J, Glöckner FO (2007). SILVA: A comprehensive online resource for quality checked and aligned ribosomal RNA sequence data compatible with ARB. Nucleic Acids Res.

[27] Glöckner FO, Yilmaz P, Quast C, Gerken J, Beccati A, Ciuprina A (2017). 25 Years of serving the community with ribosomal RNA gene reference databases and tools. J Biotechnol.

[28] Lagesen K, Hallin P, Rødland EA, Staerfeldt H, Rognes T, Ussery DW (2007). RNAmmer: consistent and rapid annotation of ribosomal RNA genes. Nucleic Acids Res.

[29] Lee JH, Yi H, Chun J (2011). rRNASelector: a computer program for selecting ribosomal RNA encoding sequences from metagenomic and metatranscriptomic shotgun libraries. J Microbiol.

[30] Eddy SR (2011). Accelerated profile HMM searches. PLOS Comput Biol.

[31] Pruesse E, Peplies J, Glöckner FO (2012). SINA: accurate high throughput multiple sequence alignment of ribosomal RNA. Bioinformatics.

[32] Nawrocki EP, Eddy SR (2013). Infernal 1.1: 100-fold faster RNA homology searches. Bioinformatics.

[33] Kalvari I, Nawrocki EP, Ontiveros-Palacios N, Argasinska J, Lamkiewicz K, Marz M (2020). Rfam 14: expanded coverage of metagenomic, viral and microRNA families. Nucleic Acids Res.

[34] Vossbrink CR, Maddox JV, Fredman S, Debrunner-Vossbrinck BA, Woese CR (1987). Ribosomal RNA sequence suggests microsporidia are extremely ancient Eukarytotes. Nature.

[35] Barandun J, Hunziker M, Vossbrink CR, Klinge S (2019). Evolutionary compaction and adaptation visualized by the structure of the dormant microsporidia ribosome. Nat Microbiol.

[36] O’Leary NA, Wright MW, Brister JR, Ciufo S, Haddad D, McVeigh R, et al. Reference sequence (RefSeq) database at NCBI: current status, taxonomic expansion, and functional annotation. Nucleic Acids Res. 2016;44:D733–45. 10.1093/nar/gkv1189 PMC4702849 26553804

[37] Edgar RC (2010). Search and clustering orders of magnitude faster than BLAST. Bioinformatics.

[38] Wheeler TJ, Eddy SR (2013). nhmmer: DNA homology search with profile HMMs. Bioinformatics.

[39] Schäffer AA, Hatcher EL, Yankie L, J R Brister LS, Karsch-Mizrachi I, Nawrocki EP. VADR: Validation and annotation of virus sequence submissions to GenBank. BMC Bioinform. 2020;21:211. 10.1186/s12859-020-3537-3 PMC7245624 32448124

[40] Schäffer AA, Nawrocki EP, Choi Y, Kitts PA, Karsch-Mizrachi I, McVeigh R (2018). VecScreen\_plus\_taxonomy: imposing a tax(onomy) increase on vector contamination screening. Bioinformatics.

[41] Nawrocki EP. The SSU-ALIGN User’s Guide; 2016. http://eddylab.org/software/ssu-align/Userguide.pdf.

[42] Liu K, Porras-Alfaro A, Kuske CR, Eichorst SA, Xie G (2012). Accurate, rapid taxonomic classification of fungal large-subunit rRNA genes. Appl Environ Microbiol.

[43] Hadziavdic K, Lekang K, Lanzen A, Jonassen I, Thompson EM (2014). Characterization of the 18S rRNA gene for designing universal eukaryotic specific primers. PLOS ONE.

[44] Altschul SF, Madden TL, Schäffer AA, Zhang J, Zhang Z, Miller W (1997). Gapped BLAST and PSI-BLAST: a new generation of protein database search programs. Nucleic Acids Res.

[45] Yarza P, Yilmaz P, Panzer K, Glöckner FO, Reich M (2017). A phylogenetic framework for the kingdom fungi based on 18S rRNA gene sequences. Mar Genom.

[46] Quast C, Pruesse E, Yilmaz P, Gerken J, Schweer T, Yarza P (2013). The SILVA ribosomal RNA gene database project: improved data processing and web-based tools. Nucleic Acids Res.

[47] Kozlov AM, Zhang J, Yilmaz P, Glöckner FO, Stamatakis A (2016). Phylogeny-aware identification and correction of taxonomically mislabeled sequences. Nucleic Acids Res.

[48] Sayers E. Entrez programming utilities help [Internet]; 2010. https://www.ncbi.nlm.nih.gov/books/NBK25501/.

[49] Eddy SR (2002). A memory-efficient dynamic programming algorithm for optimal alignment of a sequence to an RNA secondary structure. BMC Bioinform.

